# Supplementary material for: Mitochondrial peptide BRAWNIN is essential for vertebrate respiratory complex III assembly
Source: Nat Commun. 2020 Mar 11;11:1312. doi: 10.1038/s41467-020-14999-2 (PMC7066179; doi:10.1038/s41467-020-14999-2)
Supplement: Supplementary file 1 — Supplementary Information [file 41467_2020_14999_MOESM1_ESM.pdf]

Supplementary Information

Mitochondrial peptide BRAWNIN is Essential for Vertebrate Respiratory Complex III Assembly

Zhang et al.

This PDF file includes:

Supplementary Note 1
Supplementary Figs. 1 to 6
Supplementary Table 1
Supplemental References

Supplementary Note 1

sORF selection Parameters

To determine the quality and likelihood of each sORF, multiple algorithms have been developed to assess ribosomal periodicity and control for inherent biases in the Ribo-Seq workflow.

1) ORFscore [1]: The ORFscore calculates the preference of ribosome protected fragments (RPFs) to accumulate in the first frame of coding sequences. To compute the ORFscore, RPFs are counted in each frame (in - frame 0, +1 frame and +2 frame). Next, the distribution of RPFs is compared to an equally sized uniform distribution using a modified chi-squared statistic. In literature, an ORFscore of at least 6 is generally considered a good score.

2) In-frame-coverage (IFC) [2]: Percentage of nucleotides covered by in-frame situated RPFs (i.e. mapping at first base of coding triplets to the last base of the stop codon).

3) Coverage Uniformity (U) [2]: Represents how uniform the ribosome footprints are distributed over the sORF sequence. This filter ranges between -1 and 1, with either boundary indicating that all ribosomes reside in one half of the sORF. A coverage uniformity of 0 implies that the ribosomes are uniformly distributed.

4) FLOSS [3]: The FLOSS algorithm is an additional method designed to distinguish true coding from non-coding sequences based on the RPF length distribution. The FLOSS algorithm provides a score based on the comparison between the RPF length distribution in each sORF and the RPF length distribution found in canonical protein-coding sequences. Based on the FLOSS score, a classification is made representing the coding tendency of sORFs.

Mitochondrial Prediction

The first method relies on mitochondrial functional prediction based on functional enrichment of SEP co-expressed genes. First, we performed co-expression analysis where SEPs were ranked by weighted gene correlation network analysis (WGCNA) of their host gene with genes in publicly available human liver, heart and skeletal muscle RNA-seq datasets (Supplementary Fig.1a, details in Method section). Next, we performed functional enrichment analysis using gene set enrichment analysis (GSEA) of ranked lists from WGCNA. To classify each candidate as mitochondrial or non-mitochondrial, we performed multi-dimensional PCA analysis of pathway normalized enrichment scores (NES) of MitoCarta ("mito") genes and a matched number of randomly chosen

"non-mitochondrial" transcription factors. We used non-hierarchical K-means clustering, averaging over 15 clusters to obtain a mitochondrial likelihood score of each SEP candidate in each of the 3 tissues (Supplementary Fig.1a). The threshold defining mitochondrial identity in each tissue was set by maximising the predictive performance of 53 known MitoCarta SEPs against 83 known non-mitochondrial SEPs. Using this method, 64 "high confidence" SEPs were included because they scored positive in all of the tissues in which they are expressed and have not been previously characterized (Supplementary Fig.1a). This method correctly classified 73% of known training set SEPs with a false positive and negative rate of 6% and 57% respectively. Notably, the classification success drops to 60% when performed in cultured skin or endothelial cells, which have lower mitochondrial content compared to heart, muscle and liver (data not shown).

Next, we selected 72 SEPs predicted to be mitochondrial because they contained a potential "mitochondrial targeting motif". This motif can be either a transmembrane domain (TM) or signal peptide (SP) with reduced hydrophobicity, a mitochondrial targeting sequence (MTS) or twin-cysteine (2C3XC, 2C9XC, C9XC & C10XC). This combination correctly retrieves 87% of known MitoCarta SEPs but has a very high false positive rate of 49% (based on known non-mitochondrial SEPs).

The third method relies on empirical evidence of protein expression in the mitochondria. 23 thresholded SEPs with matching spectra in an LC-MS/MS dataset of purified human mitochondria [4] (details in the Method section) with unknown function were included regardless of their score in the first two measures.

SEP Screening Outcome

Of 173 SEP candidates tested, we were able to obtain a credible HA fluorescence signal from the overexpression of the open reading frames of 88 candidates (50.9%) in HeLa (Supplementary Fig. 1c), representing the experimental validation of 88 uncharacterized sORF-encoded peptides (SEPs). SEP candidates with undetectable expression levels may be unstable, require the presence of autologous UTRs for transcript stability or translational efficacy, or might be secreted upon production. These were excluded from further characterization. Notably, the highest rate of successful protein expression was from the category of annotated sORFs (88.2%). 48.5% uORF and 37.5% of lincRNA-derived SEPs can also generate stable protein. Of the 3 approaches used for mitochondrial prediction, the strongest predictor was the presence of protein motifs, namely a

transmembrane domain (TMD), with 30.5% positive prediction rate (Supplementary Fig.1d). WGCNA/GSEA had a poorer-than-expected outcome because 82.8% of GSEA-predicted candidates were uORF-derived SEPs. Since GSEA utilizes the underlying host gene for functional prediction, which cannot distinguish between the main annotated ORF and the uORF-encoded proteins, we are unable to accurately decipher the function of the uORF-derived SEP. Lastly, the presence of matching spectra in existing proteomics dataset turned out to be the poorest predictor for protein validity, potentially due to off-target identifications. This underscores the importance of unbiased, experimentally-validated screening efforts as reported in this study. By inference, the presence of matching spectra alone should not be used as definitive evidence of the validity of a SEP. Of note, validated SEPs that do not localize to the mitochondria display a wide variety of subcellular localization (Supplementary Fig.1e), suggesting their potentially diverse functions in cell biology.

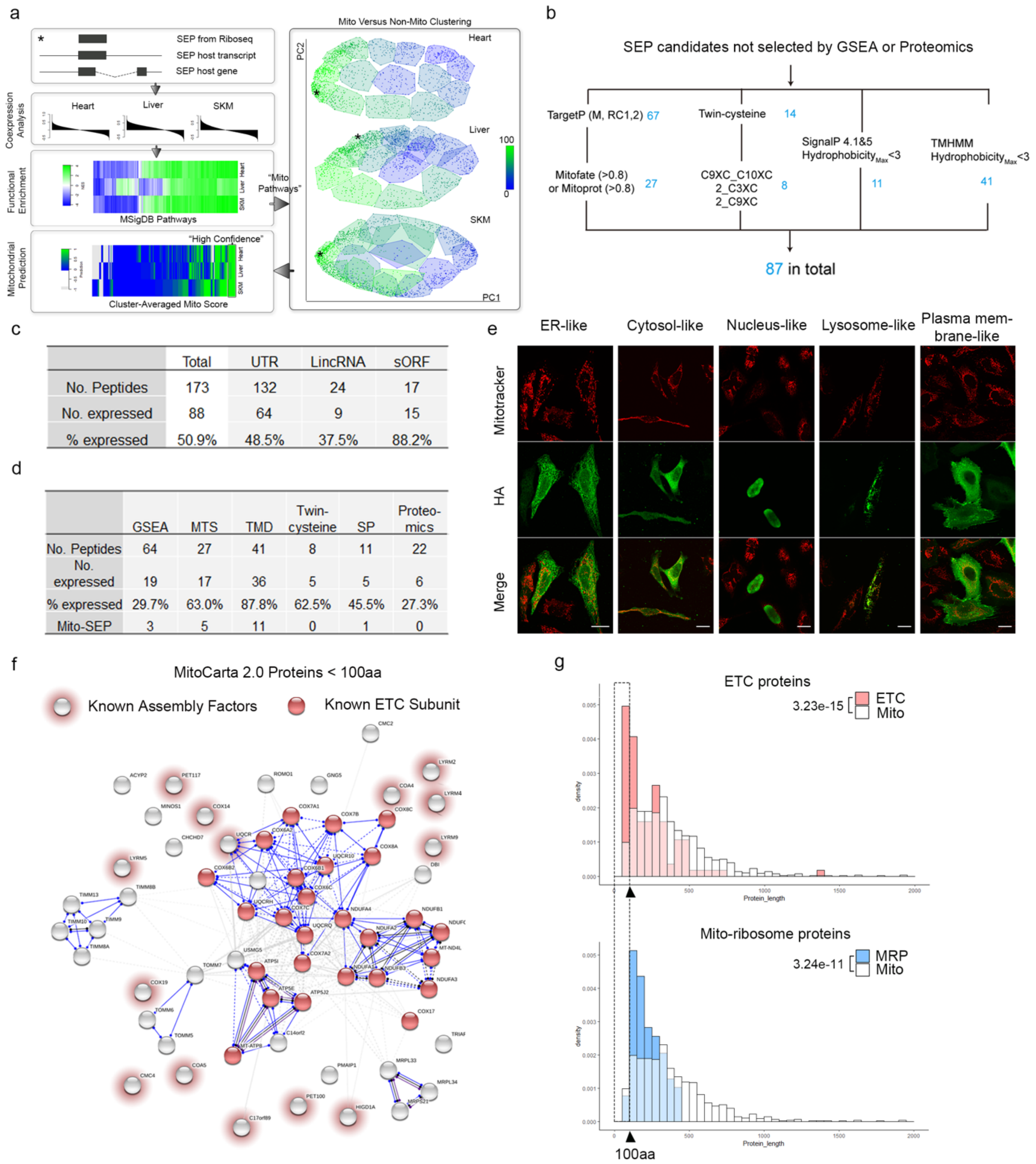

	Total	UTR	LincRNA	sORF
No. Peptides	173	132	24	17
No. expressed	88	64	9	15
% expressed	50.9%	48.5%	37.5%	88.2%

	GSEA	MTS	TMD	Twin-cysteine	SP	Proteo-mics
No. Peptides	64	27	41	8	11	22
No. expressed	19	17	36	5	5	6
% expressed	29.7%	63.0%	87.8%	62.5%	45.5%	27.3%
Mito-SEP	3	5	11	0	1	0

Supplementary Fig.1 Mito-SEP prediction pipeline identifies uncharacterized endogenous mitochondrial SEPs

a. Co-expression analysis coupled to functional enrichment analysis to identify "high confidence" SEP genes with mitochondrial gene signature. Color bar scale on the right panel refers to the percentage of known mitochondrial genes in each K-means cluster. Color bar scale on the bottom left panel refers to the K-means score. All other color bars depict normalized enrichment scores

(NES). *indicates a specific “high confidence” candidate as it transits through the pipeline. See SI text for details.

b. Workflow for identifying Mito-SEPs with potential mitochondrial targeting protein domains.

c. Numbers of screened and successfully expressed peptides (i.e. with detectable HA IF signal) according to their biotype annotation as 5’UTR, LincRNA or sORF in their genomic loci.

d. Numbers of screened and expressed peptides that were predicted by indicated methods. Gene Set Enrichment Analysis (GSEA), Mitochondrial Targeting Sequence (MTS), Trans-membrane domain (TMD), Signal Peptide (SP).

e. Representative SEP candidates with non-mitochondrial localization as revealed HA IF. Scale bar = 20 µm.

f. Human MitoCarta 2.0 proteins <100 a.a. were analyzed by STRING V10 [5] and classified according to functional ontology (GO and manual curation). Dark red circles refer to proteins with known functions in electron transport chain (ETC) complexes; light red halos refer to proteins with known role as ETC assembly factors.

g. Upper panel: Size distributions of ETC proteins (complex subunits and assembly factors, 113 proteins) and all mitochondrial proteins (Human MitoCarta 2.0). Lower panel: Size distribution of mitochondrial ribosome proteins (MRP) (78 proteins) and all mitochondrial proteins (Human MitoCarta 2.0, 1158 proteins). p-values were computed using two-sided Mann-Whitney U-test. Note the relative enrichment of ETC proteins that are below 100 a.a., compared to mito-ribosome proteins, even though components of both complexes are enriched for lower molecular weight proteins.

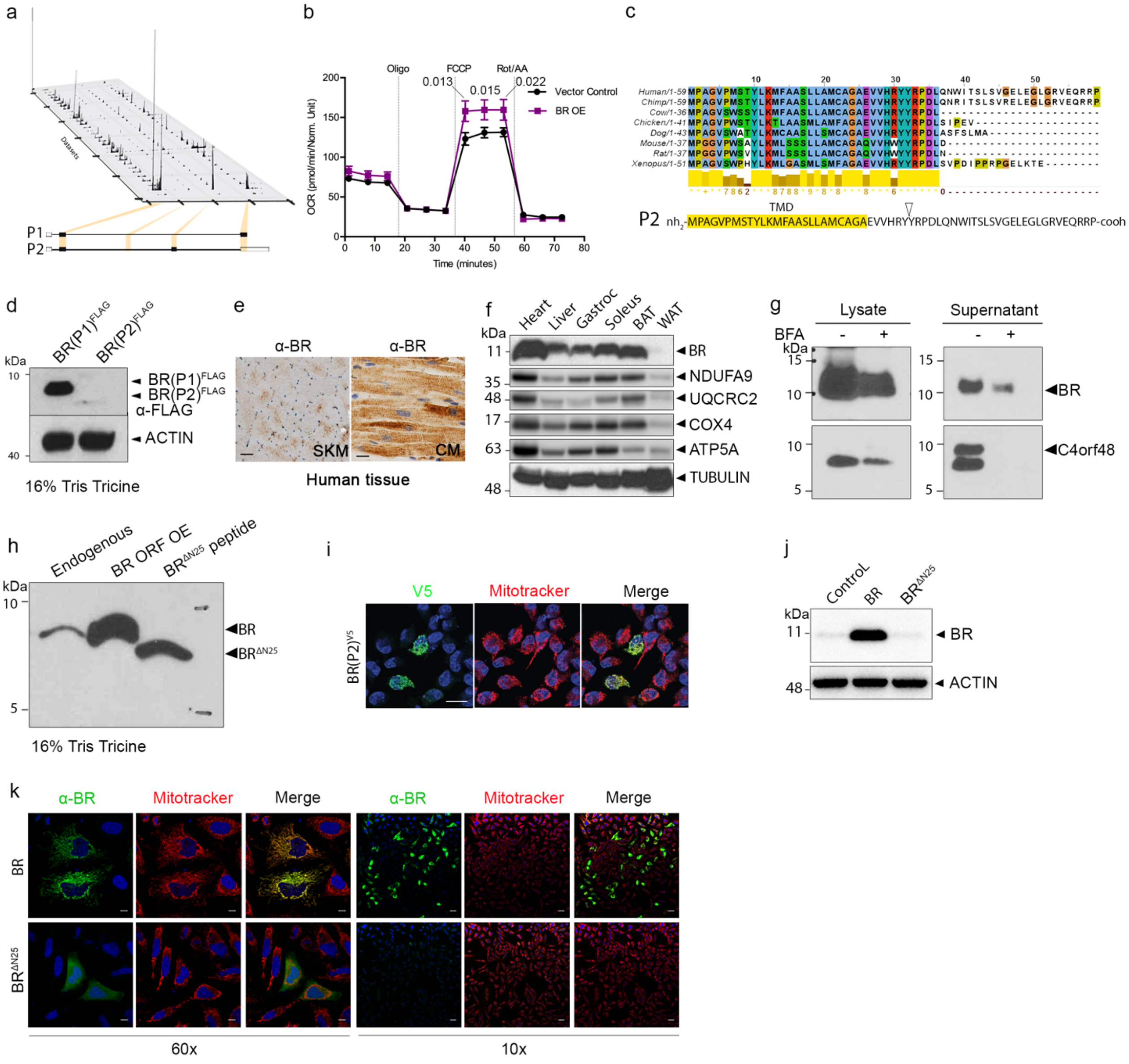

Supplementary Fig. 2 BRAWNIN (BR) is a conserved SEP at the inner mitochondrial membrane

a. Ribosome-sequencing reads from the *BRAWNIN* gene *(C12orf73)* across 31 human cell lines. Each line represents a distinct human cell line from sORFs.org.

b. Seahorse MitoStress test of U87MG cells stably transduced with a lentiviral construct expressing BR or empty vector. Oligo, oligomycin; FCCP, carbonyl cyanide 4-(trifluoromethoxy)phenylhydrazone; Rot/AA, rotenone/antimycin A. Significance levels derived from two-sided paired t-test. 6 wells of cells were examined for each treatment. Error bars indicate

SEM.

c. Alignment of the predicted BR isoform 2 (BR-P2) encoded by an alternatively spliced transcript of the *C12orf73* gene.

d. Western blot of lysates from HEK293T transfected with BR isoform 1-FLAG ($BR(P1)^{FLAG}$) and isoform 2-FLAG BR ($BR(P2)^{FLAG}$). BR-P2 (59 a.a.) is detectable but is present at much lower levels compared to BR-P1.

e. α-BR IHC in human skeletal muscle (SKM) and cardiac muscle (CM).Scale = 20 µm.

f. Western blot of endogenous mouse BR in the indicated tissue lysates. BAT = brown adipose tissue, WAT = white adipose tissue.

g. Western blot of intracellular (lysate) versus extracellular (supernatant) BR of HEK293T cells transfected with the BR ORF. C4ORF48, a bona fide secreted peptide of the same length (unpublished) is used as a control. Brefeldin A (BFA) inhibits the classical secretory pathway. Note that extracellular BR is not completely depleted by BFA as would be expected of a bona fide secreted protein.

h. Western blot of endogenous, overexpressed BR in HEK293T separated on a 16% Tris Tricine gel together with a synthetic BR peptide lacking N-terminal 25 amino acids ($BR^{\Delta N25}$).

i. BR-P2-V5 tagged protein can be detected by IF when overexpressed in HeLa and localizes to the mitochondria as indicated by colocalization with Mitotracker. Scale bar = 20 µm.

j. Western blot of HEK293T lysates transfected with full length BR and BR lacking N-terminal 25 amino acids encoding the predicted signal peptide or transmembrane domain ($BR^{\Delta N25}$).

k. IF of transiently over-expressed BR and $BR^{\Delta N25}$ with α-BR in HeLa. Scale bar = 10 µm (60X); 50 µm (10X).

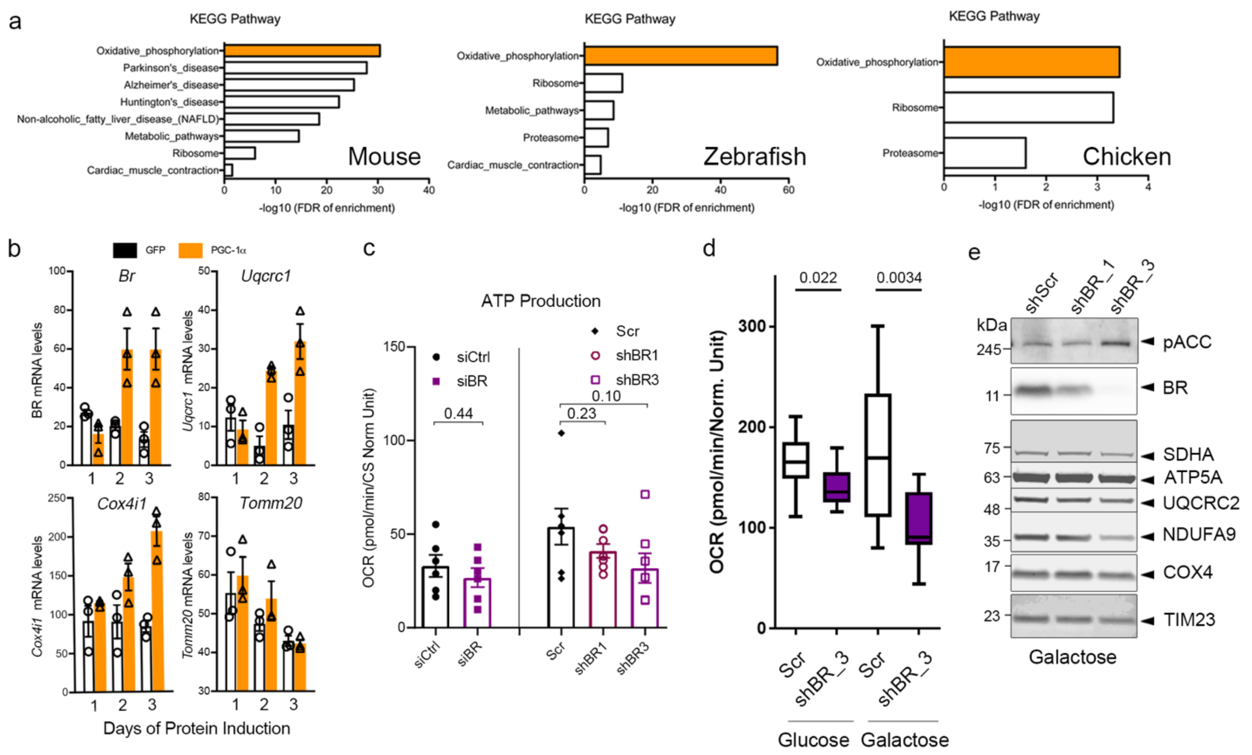

Supplementary Fig. 3 BR is an AMPK target that potentiates OXPHOS in human U87MG cells

a. Enriched KEGG pathways by Panther gene ontology analysis of the top 200 BR co-expressed genes (from COXPRESdb v7.0 [6]) in mouse, zebrafish and chicken.

b. mRNA levels of *Br* and other mitochondrial proteins in mouse myotubes with enforced expression of PGC-1α. Data are obtained from GEO omnibus dataset GDS1879 with probeset 114297_f_at for *Br*. Data from 3 independent experiments presented. Error bars indicate SEM.

c. ATP production calculated from experiment in Fig. 3d,e. Data are mean and SEM of 6 technical replicates which are representative of 2 and 3 biological replicates for shRNA and siRNA-mediated depletion, respectively. p-values from two-sided unpaired t-test. Error bars indicate SEM.

d. Basal OCR of HEK293T with stable shRNA-mediated knockdown of BR grown under normal (glucose) and oxidative (galactose) conditions. Boxes spread from the 25th to 75th percentiles with medians show in the middle. Whiskers indicate minimum to maximum values. p-values from two-sided unpaired t-test. 12 wells of cells were examined for each treatment.

e. SDS-PAGE and western blot analysis of HEK293T with stable shRNA-mediated knockdown

of BR cultured in galactose.

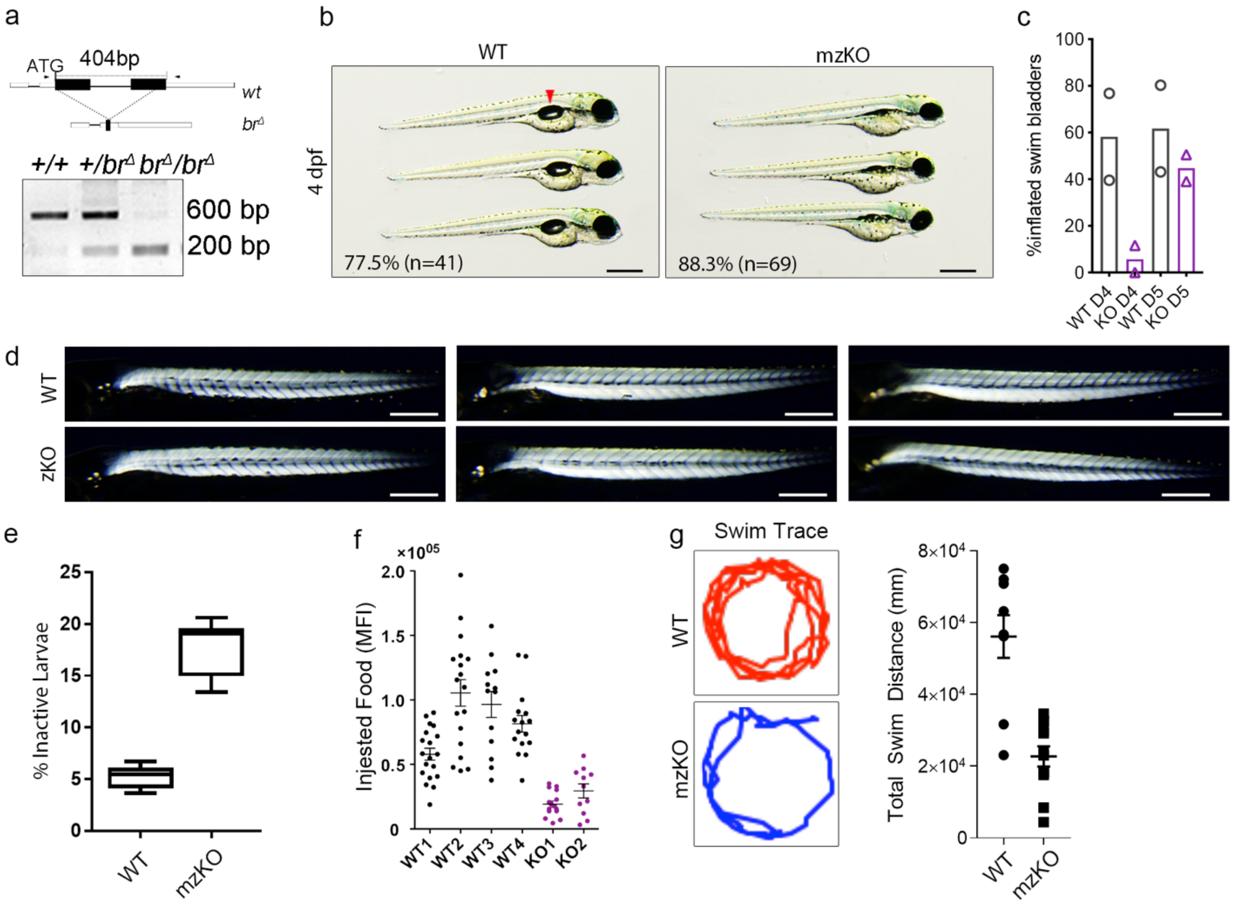

Supplementary Fig. 4 Knockout of *Br* in zebrafish causes lethal mitochondrial deficiency

a. Deletion strategy of *zf br* by Crispr/Cas9 (top); PCR of genomic locus with indicated primers spanning deletion site.

b. Brightfield images of 4 days post fertilization (dpf) WT and KO larvae. Images represent indicated percentage of all larvae analyzed with indicated n numbers. Swim bladder in WT is indicated by red arrowhead. Scale bar = 100 µm.

c. Quantification of the percentage of larvae from two independent clutches (n=41 for WT, n=69 for KO) with inflated swim bladders at the indicated age.

d. Birefringence analysis of 5 dpf WT and KO larvae to reveal skeletal muscle fiber integrity and organization. Scale bar = 150 µm.

e. Percentage of inactive larvae at 6 dpf, scored as larvae that remain motionless on the bottom of the tank for 1 minute. 40 animals monitored per genotype. Boxes extend from the 25th to 75th percentiles with medians show in the middle. Whiskers indicate minimum to maximum values.

f. Feeding behavior as measured by the amount of fluorescent paramecium ingested at 7 dpf in 4 clutches of WT and 2 clutches of mz KO larvae. MFI = mean fluorescence intensity of gut region post-feeding. Number of examined animals are 19, 18, 13, 16, 15, 11 for WT clutch 1-4 and KO clutch 1-2, respectively. Error bars indicate SEM.

g. Representative swim trace of WT versus mzKO larvae at 11 dpf (left). 9 WT and 12 mzKO larvae were examined, respectively. Quantitation of overall distance swam in a fixed assay interval (right). Error bars indicate SEM.

a

Deleted reaction		Effect Size Lactate	Succinate	Total
O2tm	O2 transport into Mitochondria	15.107	0.619	15.726
O2t	O2 uptake by cell	15.022	0.448	15.469
CIV_MitoCore	Complex IV (ETC)	11.527	3.645	15.171
CIII_MitoCore	Complex III (ETC)	11.254	3.558	14.812
PDHm	Pyruvate dehydrogenase	4.817	0.496	5.313
L_LACt2r	Lactate uptake by cell	2.654	0.067	2.721
LDH_L	Lactate dehydrogenase	2.654	0.024	2.679
Hct_MitoCore	Proton transport	0.217	1.763	1.980

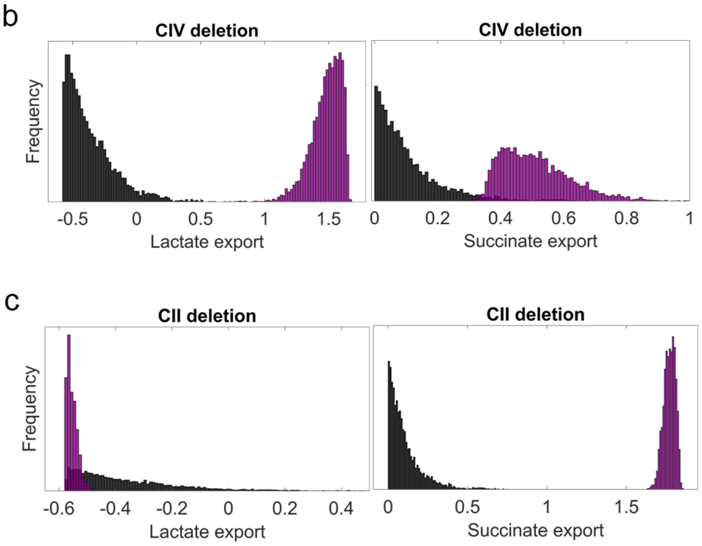

Supplementary Fig. 5 Br functionally and physically interacts with electron transport chain Complex III

a. MitoCore reactions, ranked by how strongly their shutdown would increase both lactate and succinate export. (see SI for details).

b. Distributions of lactate and succinate export flux levels in 5000 random samples under unconstrained or CIV-flux-constrained.

c. Distributions of lactate and succinate export flux levels in 5000 random samples under unconstrained or CII-flux-constrained.

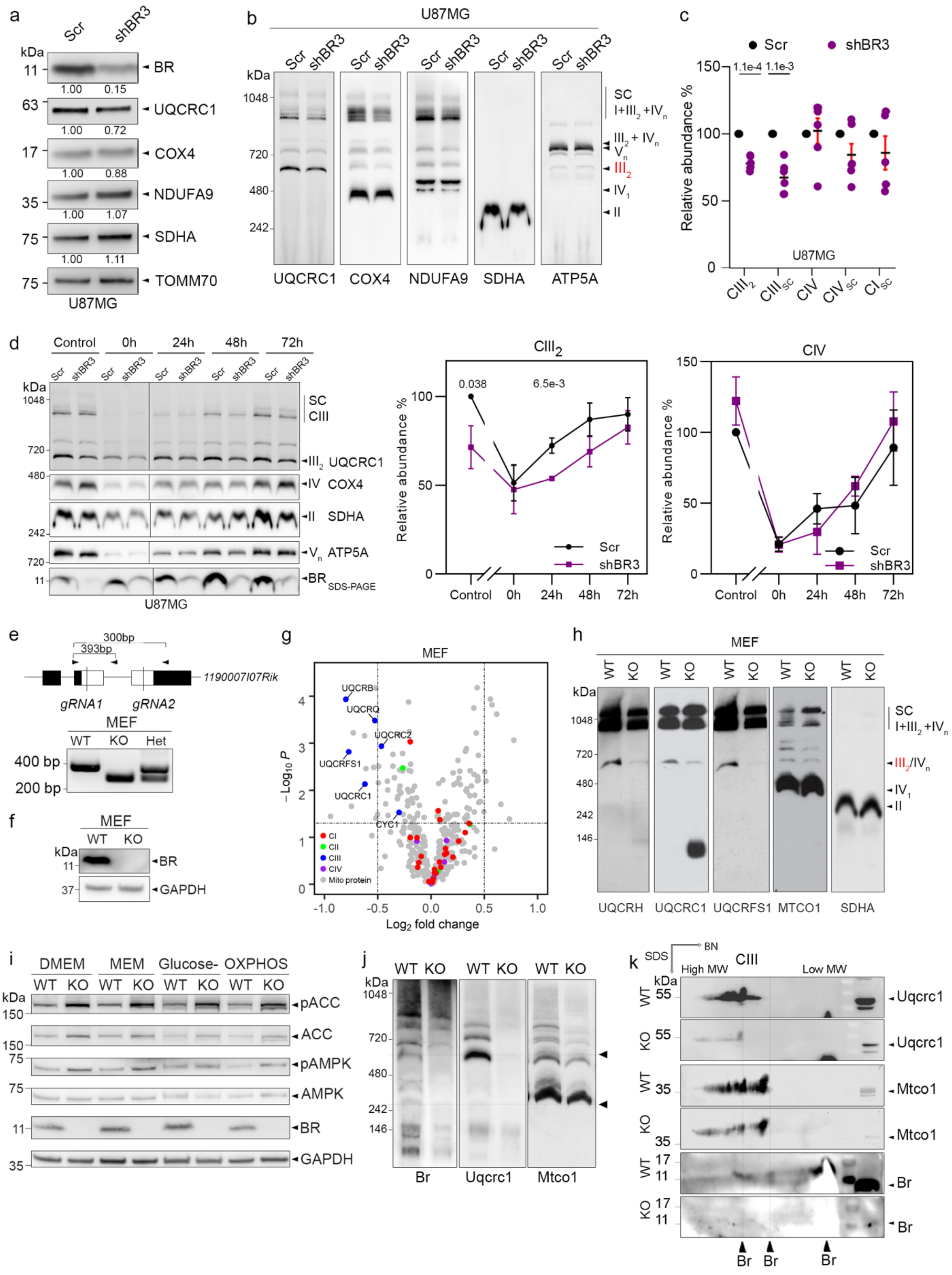

Supplementary Fig. 6 Br is required for Complex III Assembly and Activity

a. SDS-PAGE of mitochondria from control and *sh*BR3 U87MG. Protein abundance was normalized to the level of TOMM70.

b. BN-PAGE of mitochondria from U87MG stably transduced with scramble control (Scr) or shRNA targeting *BR* (shBR3).

c. Relative abundance of $CIII_2$, CIII in supercomplex ($CIII_{SC}$), CIV, CIV in supercomplex (CIV_{SC}), CI in supercomplex (CI_{SC}) in scramble control (Scr) or shRNA targeting *BR* (shBR3) transduced U87MG cells. Levels in Scr control were set to 1 in normalization. Error bars indicate SEM of five independent replicates. p-value from two-sided paired t-test.

d. BN-PAGE of mitochondria from U87MG cells after 8 days of doxycycline treatment at 15 µg/mL. Mitochondria were harvested at indicated times after releasing the doxycycline treatment. Relative abundance of $CIII_2$ and CIV is shown in the right panel. Error bars indicate SEM of three independent replicates. p-value from two-sided unpaired t-test.

e. Crispr/Cas9 mediated KO of Br (*1190007I07Rik*) using two gRNAs targeting the coding region.

f. Immunoblotting validation of Br knock-out in mouse embryonic fibroblast (MEF).

g. Quantitative proteomic analysis of WT and *Br* KO MEF. Mean values of $\log_2$ fold change (*Br* KO/WT) of detected mitochondrial protein plotted. Proteins of ETC complexes are labeled in the indicated colors. CI-CIV = Complex I - Complex IV.

h. BN-PAGE analysis of isolated MEF mitochondria.

i. SDS-PAGE analysis of total and phosphorylated ACC and AMPK level in WT and *Br* KO MEF.

j. BN-PAGE and western blot analysis with zebrafish-specific Br antibody. Position of Br is indicated by black arrowheads as these signals are completely absent in the KO. Membrane was completely stripped and reprobed with Uqcrc1 and Mtco1 to reveal the position of CIII and CIV respectively. These data are representative of 4 independent BN-PAGE experiments.

k. 2nd dimension SDS-PAGE analysis to confirm that the indicated signal in (i) is indeed specific to Br, as judged by a signal at the expected 10 kD in the WT which is absent in the KO. A sub-population of zebrafish Br co-migrates with CIII.

Supplementary Table 1. MitoCore: Individual deletions of CIII and CIV genes explain metabolomic observations.

Top genes ranked by their sum of effect size on lactate and succinate fluxes upon deletion. Highlighted in green are genes from complexes III and IV, in orange are genes from complex I.

			Effect Size		
**Rank**	**Gene Symbol**	**Gene Name**	**Lactate**	**Succinate**	**Sum**
1	UQCRFS1	ubiquinol-cytochrome c reductase, Rieske iron-sulfur polypeptide 1	11.772	3.712	15.485
2	COX4I2	cytochrome c oxidase subunit 4I2	11.759	3.653	15.412
3	CYC1	cytochrome c1	11.718	3.689	15.408
4	COX4I1	cytochrome c oxidase subunit 4I1	11.657	3.717	15.374
5	MT-CO1	mitochondrially encoded cytochrome c oxidase I	11.675	3.695	15.370
6	COX8C	cytochrome c oxidase subunit 8C	11.703	3.646	15.349
7	COX5A	cytochrome c oxidase subunit 5A	11.679	3.635	15.314
8	CYCS	cytochrome c, somatic	11.660	3.651	15.311
9	COX6A2	cytochrome c oxidase subunit 6A2	11.699	3.606	15.304
10	COX6C	cytochrome c oxidase subunit 6C	11.586	3.678	15.264
11	UQCRH	ubiquinol-cytochrome c reductase hinge protein	11.539	3.719	15.258
12	COX5B	cytochrome c oxidase subunit 5B	11.541	3.683	15.223
13	COX7A2	cytochrome c oxidase subunit 7A2	11.559	3.629	15.188
14	COX8A	cytochrome c oxidase subunit 8A	11.495	3.656	15.151
15	TTC19	tetratricopeptide repeat domain 19	11.510	3.623	15.133
16	COX7B	cytochrome c oxidase subunit 7B	11.480	3.633	15.113
17	UQCRC2	ubiquinol-cytochrome c reductase core protein 2	11.413	3.657	15.070
18	UQCR10	ubiquinol-cytochrome c reductase, complex III subunit X	11.454	3.614	15.068
19	COX7C	cytochrome c oxidase subunit 7C	11.386	3.651	15.038
20	UQCRB	ubiquinol-cytochrome c reductase binding protein	11.377	3.592	14.969
21	MT-CO3	mitochondrially encoded cytochrome c oxidase III	11.311	3.641	14.952
22	COX7A1	cytochrome c oxidase subunit 7A1	11.339	3.594	14.933
23	MT-CO2	mitochondrially encoded cytochrome c oxidase II	11.384	3.541	14.925
24	COX7A2L	cytochrome c oxidase subunit 7A2 like	11.257	3.664	14.921
25	COX6B2	cytochrome c oxidase subunit 6B2	11.203	3.597	14.801
26	UQCR11	ubiquinol-cytochrome c reductase, complex III subunit XI	11.308	3.492	14.800
27	COX6A1	cytochrome c oxidase subunit 6A1	11.232	3.520	14.752
28	MT-CYB	mitochondrially encoded cytochrome b	11.225	3.498	14.722
29	COX6B1	cytochrome c oxidase subunit 6B1	11.018	3.567	14.585
30	UQCRC1	ubiquinol-cytochrome c reductase core protein 1	10.970	3.422	14.392
31	DLD	dihydrolipoamide dehydrogenase	5.113	0.249	5.363
32	DLAT	dihydrolipoamide S-acetyltransferase	4.867	0.452	5.320
33	PDHB	pyruvate dehydrogenase E1 beta subunit	4.846	0.401	5.247
34	PDHA1	pyruvate dehydrogenase E1 alpha 1 subunit	4.406	0.407	4.813
35	GOT2	glutamic-oxaloacetic transaminase 2	1.275	0.042	1.317
36	NDUFA9	NADH:ubiquinone oxidoreductase subunit A9	1.025	0.259	1.284
37	MDH1	malate dehydrogenase 1	1.179	0.060	1.239
38	NDUFA3	NADH:ubiquinone oxidoreductase subunit A3	0.934	0.263	1.197
39	NDUFA11	NADH:ubiquinone oxidoreductase subunit A11	0.950	0.211	1.160
40	NDUFS6	NADH:ubiquinone oxidoreductase subunit S6	1.030	0.110	1.140
41	MT-ND1	mitochondrially encoded NADH:ubiquinone oxidoreductase core subunit 1	1.016	0.123	1.139
42	NDUFS8	NADH:ubiquinone oxidoreductase core subunit S8	1.007	0.109	1.116
43	NDUFA5	NADH:ubiquinone oxidoreductase subunit A5	0.982	0.122	1.104
44	NDUFS2	NADH:ubiquinone oxidoreductase core subunit S2	0.952	0.124	1.076
45	NDUFB10	NADH:ubiquinone oxidoreductase subunit B10	0.965	0.103	1.068
46	NDUFA8	NADH:ubiquinone oxidoreductase subunit A8	0.992	0.070	1.063
47	NDUFB4	NADH:ubiquinone oxidoreductase subunit B4	0.951	0.094	1.045
48	NDUFB9	NADH:ubiquinone oxidoreductase subunit B9	0.974	0.068	1.042
49	NDUFC2	NADH:ubiquinone oxidoreductase subunit C2	1.028	0.014	1.042
50	NDUFA13	NADH:ubiquinone oxidoreductase subunit A13	0.951	0.088	1.039
51	MT-ND3	mitochondrially encoded NADH:ubiquinone oxidoreductase core subunit 3	0.946	0.093	1.038

			Effect Size		
Rank	Gene Symbol	Gene Name	Lactate	Succinate	Sum
52	NDUFS1	NADH:ubiquinone oxidoreductase core subunit S1	1.028	0.006	1.034
53	NDUFA12	NADH:ubiquinone oxidoreductase subunit A12	0.941	0.092	1.033
54	NDUFA6	NADH:ubiquinone oxidoreductase subunit A6	0.995	0.035	1.030
55	NDUFC1	NADH:ubiquinone oxidoreductase subunit C1	0.952	0.077	1.029
56	NDUFB1	NADH:ubiquinone oxidoreductase subunit B1	0.978	0.051	1.029
57	NDUFB2	NADH:ubiquinone oxidoreductase subunit B2	0.925	0.103	1.028
58	NDUFA4	NDUFA4 mitochondrial complex associated	0.955	0.061	1.017
59	NDUFA4L2	NDUFA4 mitochondrial complex associated like 2	0.911	0.101	1.012
60	NDUFS3	NADH:ubiquinone oxidoreductase core subunit S3	0.965	0.045	1.010
61	MT-ND4	mitochondrially encoded NADH:ubiquinone oxidoreductase core subunit 4	0.941	0.061	1.002
62	NDUFA2	NADH:ubiquinone oxidoreductase subunit A2	0.968	0.019	0.988
63	NDUFS7	NADH:ubiquinone oxidoreductase core subunit S7	0.928	0.059	0.987
64	MT-ND6	mitochondrially encoded NADH:ubiquinone oxidoreductase core subunit 6	0.932	0.055	0.987
65	NDUFA7	NADH:ubiquinone oxidoreductase subunit A7	0.912	0.060	0.973
66	NDUFAB1	NADH:ubiquinone oxidoreductase subunit AB1	0.938	0.027	0.965
67	NDUFA10	NADH:ubiquinone oxidoreductase subunit A10	0.930	0.029	0.959
68	MT-ND4L	mitochondrially encoded NADH:ubiquinone oxidoreductase core subunit 4L	0.910	0.030	0.941
69	NDUFB7	NADH:ubiquinone oxidoreductase subunit B7	0.931	0.004	0.934
70	MECR	mitochondrial trans-2-enoyl-CoA reductase	0.072	0.172	0.244
71	ACOT2	acyl-CoA thioesterase 2	0.153	0.075	0.228
72	FECH	ferrochelatase	0.029	0.182	0.211
73	MTHFD2L	methylenetetrahydrofolate dehydrogenase (NADP+ dependent) 2 like	0.048	0.153	0.201
74	GPD1	glycerol-3-phosphate dehydrogenase 1	0.042	0.139	0.182
75	NNT	nicotinamide nucleotide transhydrogenase	0.056	0.125	0.181
76	UCP2	uncoupling protein 2	0.046	0.134	0.180
77	ACO2	aconitase 2	0.158	0.020	0.178
78	ACSS1	acyl-CoA synthetase short chain family member 1	0.165	0.012	0.176
79	AADAT	aminoadipate aminotransferase	0.019	0.132	0.150
80	AMT	aminomethyltransferase	0.010	0.138	0.148
81	PPA2	pyrophosphatase (inorganic) 2	0.073	0.073	0.146
82	QDPR	quinoid dihydropteridine reductase	0.010	0.135	0.145
83	ALDH18A1	aldehyde dehydrogenase 18 family member A1	0.070	0.068	0.138
84	AOC1	amine oxidase copper containing 1	0.044	0.079	0.122
85	ODC1	ornithine decarboxylase 1	0.068	0.050	0.118
86	ATP8B2	ATPase phospholipid transporting 8B2	0.031	0.087	0.118
87	DHFR	dihydrofolate reductase	0.031	0.080	0.111
88	ATP11B	ATPase phospholipid transporting 11B (putative)	0.085	0.026	0.110
89	MPST	mercaptopyruvate sulfurtransferase	0.017	0.092	0.109
90	MCEE	methylmalonyl-CoA epimerase	0.033	0.074	0.107
91	GPD1L	glycerol-3-phosphate dehydrogenase 1 like	0.020	0.084	0.104
92	HAL	histidine ammonia-lyase	0.063	0.032	0.095
93	PTPMT1	protein tyrosine phosphatase mitochondrial 1	0.007	0.080	0.087
94	MAT2A	methionine adenosyltransferase 2A	0.077	0.010	0.087
95	HGD	homogentisate 1,2-dioxygenase	0.026	0.058	0.084
96	PGAM2	phosphoglycerate mutase 2	0.035	0.038	0.073
97	ADSSL1	adenylosuccinate synthase like 1	0.016	0.054	0.069
98	MCCC2	methylcrotonoyl-CoA carboxylase 2	0.023	0.046	0.069
99	AK3	adenylate kinase 3	0.009	0.060	0.069
100	MAOB	monoamine oxidase B	0.020	0.034	0.054
101	PCCA	propionyl-CoA carboxylase subunit alpha	0.024	0.028	0.052
102	UCP3	uncoupling protein 3	0.032	0.011	0.043

Supplementary References

1. Bazzini, A. A. *et al.* Identification of small ORFs in vertebrates using ribosome footprinting and evolutionary conservation. *EMBO J.* **33**, 981–993 (2014).
2. Olexiouk, V., Van Criekinge, W. & Menschaert, G. An update on sORFs.org: a repository of small ORFs identified by ribosome profiling. *Nucleic Acids Res.* **46**, D497–D502 (2018).
3. Ingolia, N. T. *et al.* Ribosome Profiling Reveals Pervasive Translation Outside of Annotated Protein-Coding Genes. *Cell Rep.* **8**, 1365–1379 (2014).
4. Stroud, D. A. *et al.* Accessory subunits are integral for assembly and function of human mitochondrial complex I. *Nature* **538**, 123–126 (2016).
5. Szklarczyk, D. *et al.* STRING v10: protein–protein interaction networks, integrated over the tree of life. *Nucleic Acids Res.* **43**, D447–D452 (2015).
6. Obayashi, T., Kagaya, Y., Aoki, Y., Tadaka, S. & Kinoshita, K. COXPRESdb v7: a gene coexpression database for 11 animal species supported by 23 coexpression platforms for technical evaluation and evolutionary inference. *Nucleic Acids Res.* **47**, D55–D62 (2019).
