# Supplementary material for: Mitochondrial peptide BRAWNIN is essential for vertebrate respiratory complex III assembly
Source: Nat Commun. 2020 Mar 11;11:1312. doi: 10.1038/s41467-020-14999-2 (PMC7066179; doi:10.1038/s41467-020-14999-2)
Supplement: Supplementary file 8 — Reporting Summary [file 41467_2020_14999_MOESM8_ESM.pdf]

nature research

Corresponding author(s): Lena Ho

Last updated by author(s): 08/Feb/2019

Reporting Summary

Nature Research wishes to improve the reproducibility of the work that we publish. This form provides structure for consistency and transparency in reporting. For further information on Nature Research policies, see Authors & Referees and the Editorial Policy Checklist.

Statistics

For all statistical analyses, confirm that the following items are present in the figure legend, table legend, main text, or Methods section.

n/a	Confirmed	
☐	☒	The exact sample size (*n*) for each experimental group/condition, given as a discrete number and unit of measurement
☐	☒	A statement on whether measurements were taken from distinct samples or whether the same sample was measured repeatedly
☐	☒	The statistical test(s) used AND whether they are one- or two-sided<br>*Only common tests should be described solely by name; describe more complex techniques in the Methods section.*
☒	☐	A description of all covariates tested
☒	☐	A description of any assumptions or corrections, such as tests of normality and adjustment for multiple comparisons
☐	☒	A full description of the statistical parameters including central tendency (e.g. means) or other basic estimates (e.g. regression coefficient) AND variation (e.g. standard deviation) or associated estimates of uncertainty (e.g. confidence intervals)
☐	☒	For null hypothesis testing, the test statistic (e.g. *F*, *t*, *r*) with confidence intervals, effect sizes, degrees of freedom and *P* value noted<br>*Give P values as exact values whenever suitable.*
☒	☐	For Bayesian analysis, information on the choice of priors and Markov chain Monte Carlo settings
☒	☐	For hierarchical and complex designs, identification of the appropriate level for tests and full reporting of outcomes
☒	☐	Estimates of effect sizes (e.g. Cohen's *d*, Pearson's *r*), indicating how they were calculated

Our web collection on statistics for biologists contains articles on many of the points above.

Software and code

Policy information about availability of computer code

Data collection	Confocal images were collected using Olympus FV3000.<br>Fluorescence blots were scanned using Licor Image Studio.<br>Chemiluminescence was captured by X-ray films (Santa-Cruz) or a Chemidoc imaging system (Bio-Rad).<br>MitoStress test data were obtained using XF96 Seahorse Wave software (Agilent).<br>Images used for zebrafish motility tracing were taken using a Basler USB3 camera.<br>Optical density was recorded with a Tecan Microplate Reader M200 (Tecan).
Data analysis	Weighted Correlation Network Analysis (WGCNA) was performed with the WGCNA package in R.<br>Normalized enrichment score (NES) in gene set enrichment analysis (GSEA) was analyzed with the fgsea package in R.<br>Mitochondrial targeting motifs were predicted using the online sites of TargetP, Mitoprot, TMHMM and SignalP (4.1 and 5).<br>SearchGui tool, X!tandem, MSGF+ and PeptideShaker were used in peptide mass spectrum mapping.<br>ZiFiT was used to design Crispr gene knock-out guide RNA.<br>The background subtraction algorithms of Python utilizing OpenCV library was used in zebrafish motility monitoring.<br>The CHRR algorithm of Cobra toolbox 3.0 was used in the MitoCore modeling.<br>Fluorescence blots were quantified using Licor Image Studio.<br>Chemiluminescence blots were quantified using Bio-RAD Image Lab 6.0.1.<br>Statistical tests and data visualization were performed with Graph Pad and R using publicly available packages cited in the Methods.<br>R code for mitochondria prediction by WGCNA/GSEA method is available at https://github.com/LenaHoLab/Zhang-et-al-manuscript-R-code.

For manuscripts utilizing custom algorithms or software that are central to the research but not yet described in published literature, software must be made available to editors/reviewers. We strongly encourage code deposition in a community repository (e.g. GitHub). See the Nature Research guidelines for submitting code & software for further information.

Data

Policy information about availability of data

All manuscripts must include a data availability statement. This statement should provide the following information, where applicable:

- Accession codes, unique identifiers, or web links for publicly available datasets
- A list of figures that have associated raw data
- A description of any restrictions on data availability

All datasets and accession codes generated during in this current study are will be available upon publication with no restrictions.
Proteomics data are available from Proteome Xchange. Accession: PXD016718, [http://proteomecentral.proteomexchange.org/cgi/GetDataset?ID=PXD016718].
Source data for all western blots shown are available as a manuscript supplement ,10.6084/m9.figshare.11406999, [https://figshare.com/s/76fbb5bf11af6d5d01af].
Weblinks of publicly available datasets that used in the study:
[http://www.sorfs.org/]
Skeletal muscle dataset for GSEA: [https://www.ncbi.nlm.nih.gov/geo/query/acc.cgi?acc=GSE120862]
Skin data for GSEA:
[https://www.ncbi.nlm.nih.gov/geo/query/acc.cgi?acc=GSE85861]
Liver dataset for GSEA:
[https://www.ncbi.nlm.nih.gov/geo/query/acc.cgi?acc=GSE94660]
Heart dataset for GSEA:
[https://www.ebi.ac.uk/ega/studies/EGAS00001002454]

Field-specific reporting

Please select the one below that is the best fit for your research. If you are not sure, read the appropriate sections before making your selection.

☒ Life sciences ☐ Behavioural & social sciences ☐ Ecological, evolutionary & environmental sciences

For a reference copy of the document with all sections, see nature.com/documents/nr-reporting-summary-flat.pdf

Life sciences study design

All studies must disclose on these points even when the disclosure is negative.

Sample size	The sample size of experiments are disclosed in the figure legend and commonly accepted samples sizes were used. In zebrafish related experiments, sample sizes were estimated based on similar experiments preformed in the past.
Data exclusions	No data was excluded from the study.
Replication	Quantitative experiments were replicated multiple times as indicated in the figure legends and method. When representative data were shown, numbers of replicates are as follow: Experiments of Fig. 1e, 2e, 2f, 2i, 2j, 2k,3b,4a, 5f, 6f, Supplementary Fig.1e, Supplementary Fig.2d, Supplementary Fig.2e, Supplementary Fig.2g, Supplementary Fig.2h, Supplementary Fig.2i, Supplementary Fig.2j, Supplementary Fig.2k, Supplementary Fig.4b, Supplementary Fig.4d, Supplementary Fig.6f, Supplementary Fig.6h, Supplementary Fig.6i, Supplementary Fig.6j, Supplementary Fig.6k were repeated independently for two times with similar data obtained in all replicates. Experiments of Fig. 2c, 2d, 2g, 2h, 2l, 2m, 3c, 4b, 5e, Supplementary Fig.2f, Supplementary Fig.3e, Supplementary Fig.6a, Supplementary Fig.6e were repeated independently for three times with similar data obtained in all replicates. Experiments of Fig. 4e, Supplementary Fig.4a, Supplementary Fig.6b were repeated independently for more than three times with similar data obtained in all replicates. Where possible, experimental replicates were performed by different researchers independently.
Randomization	Randomization is not involved in this study as there were no different treatments imposed on the same cohort of animals.
Blinding	The experiments were not blinded as quantitative data were collected by equipments and softwares.

Reporting for specific materials, systems and methods

We require information from authors about some types of materials, experimental systems and methods used in many studies. Here, indicate whether each material, system or method listed is relevant to your study. If you are not sure if a list item applies to your research, read the appropriate section before selecting a response.

Materials & experimental systems

n/a	Involved in the study
☐	☒ Antibodies
☐	☒ Eukaryotic cell lines
☒	☐ Palaeontology
☐	☒ Animals and other organisms
☒	☐ Human research participants
☒	☐ Clinical data

Methods

n/a	Involved in the study
☒	☐ ChIP-seq
☒	☐ Flow cytometry
☒	☐ MRI-based neuroimaging

Antibodies

Antibodies used

Following antibodies were used in immunoblotting: anti-BR, 1:3000 (Novus, NBP1-90536); custom anti-human BR (1 μg/ml); custom anti-zebrafish BR (1 μg/ml); anti-ACTIN, 1:5000 (Sigma, A2228); anti-TOMM20 , 1:5000 (Proteintech, 11802-1-AP); anti-CS, 1:2000 (Santa Cruz, SC-390693); anti-ATP5A, 1:5000 (Santa Cruz, SC-136178); anti-TOMM70, 1:5000 (AbClonal, A4349); anti-VDAC1 , 1:2000 (AbClonal, A0810); anti-MTCOI, 1:3000 (Abcam, ab14705); anti-TIM23, 1:5000 (Proteintech, 11123-1-AP); anti-OXPHOS cocktail, 1:5000 (Invitrogen, 45-7999); anti-UQCRC1, 1:5000 (AbClonal, A3339); anti-NDUFA9, 1:3000 (AbClonal, A3196); anti-SDHA, 1:5000 (AbClonal, A2594); anti-TUBULIN, 1:5000 (Sigma, T9822); anti-ACC, 1:2000 (CST, 3662S); anti-pACC, 1:2000 (CST, 3661S); anti-GAPDH, 1:5000 (CST, 2118S); anti-FLAG, 1:2000 (BioLegend, 637302); anti-C4ORF48 (Abcam, ab185315); anti-COX4, 1:3000 (AbClonal, A10098); anti-UQCRC2, 1:5000 (Abclonal, A4181); anti-UQCRFS1, 1:2500 (Abcam, Ab14746); anti-UQCRH, 1:2500 (Abcam, ab134949); anti-UQCRB, 1:2000 (Sigma, HPA043060), Anti-MTCYB, 1:2000 (Proteintech, 55090-1-AP); Anti-AMPK, 1:3000 (Bio-Rad, VMA00246); Anti-pAMPK, 1:3000 (CST, 2535); HRP conjugated Goat Anti-Rabbit IgG, 1:5000 (Jackson ImmunoResearch, 111-035-003); HRP-conjugated Donkey Anti-Mouse IgG,1:5000 (Jackson ImmunoResearch,715-035-150), HRP-conjugated Donkey Anti-Rat IgG, 1:5000 (Jackson ImmunoResearch, 712-035-153), IRDye 800CW goat anti-rabbit (Licor, 925-32211) and IRDye 680RD goat anti-mouse (Licor, 926-68070) .
Following antibodies were used in immunostaining: anti-BR, 1:200 (Novus, NBP1-90536); anti-HA, 1:500 (BioLegend, 901502); anti-CYTC, 1:500 (Santa Cruz, sc-13561); anti-CS, 1:500 Santa Cruz, SC-390693); anti-TOMM20, 1:500 (Proteintech, 11802-1-AP), Alexa fluro 488 goat anti-mouse IgG (Invitrogen, A11001), Alexa fluro 594 goat anti-mouse IgG (Invitrogen, A11005), Alexa fluro 647 goat anti-mouse IgG (Invitrogen, A21235), Alexa fluro 488 goat anti-rabbit IgG (Invitrogen, A11008), Alexa fluro 594 goat anti-rabbit IgG (Invitrogen, A11012), Alexa fluro 647 goat anti-rabbit IgG (Invitrogen, A21245). Secondary antibodies were used at 0.4 μg/ml.

Validation

All the BR antibodies were knock-down and knock-out validated by western in this study.
Commercial antibodies with credible validations and literature citations were used. The molecular weights of specific bands were cross-validated in literature. Molecular weight sizes of bands of interest are duly reported in the figures.
anti-ACTIN antibody (Sigma, A2228) was used for the immunoblotting of human cell samples. This antibody has been tested for western using human samples by the manufacturer (https://www.sigmaaldrich.com/catalog/product/sigma/a2228?lang=en&region=SG).
anti-TOMM20 antibody (Proteintech, 11802-1-AP) is a KO/KD validated antibody that reactive to human and zebrafish TOMM20 in western (https://www.ptglab.com/products/TOM20-Antibody-11802-1-AP.htm). It was used for the same applications in this study.
anti-CS antibody (Santa Cruz, SC-390693) was used to detect human CS protein in western and mouse protein in immunofluorescence. Both applications were validated by Santa Cruz (https://www.scbt.com/p/citrate-synthase-antibody-g-3).
anti-ATP5A antibody (Santa Cruz, SC-136178) was used to detect human protein in western. This application was validated by Santa Cruz (https://www.scbt.com/p/atp5a-antibody-51).
anti-TOMM70 antibody (AbClonal, A4349) was used for human protein samples in western, and this application was validated by Abclonal (https://abclonal.com/catalog-antibodies/TOM70PolyclonalAntibody/A4349).
anti-VDAC1 antibody (AbClonal, A0810) was used for human protein samples in western, and this application was validated by Abclonal (https://abclonal.com/catalog-antibodies/VDAC1PolyclonalAntibody/A0810).
anti-MTCOI antibody (Abcam, ab14705) was used to detect human and zebrafish MTCOI in western. This antibody was validated by Abcam in both species (https://www.abcam.com/mtco1-antibody-1d6e1a8-ab14705.html).
anti-TIM23 antibody (Proteintech, 11123-1-AP) was used to detect human TIM23 in western. This application was validated by Proteintech using HepG2 cells (https://www.ptglab.com/products/TIMM23-Antibody-11123-1-AP.htm).
anti-OXPHOS antibody cocktail (Invitrogen, 45-7999) was used for human and mouse sample western. These applications were validated by the manufacturer (https://www.thermofisher.com/antibody/product/OxPhos-Blue-Native-WB-Antibody-clone-Cocktail-Cocktail/45-7999).
anti-UQCRC1 antibody (AbClonal, A3339) was used to probe human, mouse and zebrafish UQCRC1 in western. This antibody was validated for human and mouse samples in western by Abclonal (https://abclonal.com/catalog-antibodies/UQCRC1PolyclonalAntibody/A3339). This antibody detected distinctive Complex III bands at expected sizes in zebrafish BN-PAGE western, suggesting cross-reactivity to zebrafish Uqcrc1.
anti-NDUFA9 antibody (AbClonal, A3196) was used to probe human, mouse and zebrafish UQCRC1 in western. This antibody was validated for human and mouse samples in western by Abclonal (https://abclonal.com/catalog-antibodies/NDUFA9PolyclonalAntibody/A3196). This antibody detected distinctive Complex I bands at expected sizes in zebrafish BN-PAGE western, suggesting cross-reactivity to zebrafish Ndufa9.
anti-SDHA antibody (AbClonal, A2594) was used to detect human, mouse, zebrafish SDHA in western. This antibody was validated for human and mouse samples in western by Abclonal (https://abclonal.com/catalog-antibodies/SDHAPolyclonalAntibody/A2594). This antibody detected a band at expected size of Complex II in zebrafish BN-PAGE western.
anti-TUBULIN antibody (Sigma, T9822, https://www.sigmaaldrich.com/catalog/product/sigma/t9822?lang=en&region=SG); anti-pACC antibody (CST, 3661S, https://www.cellsignal.com/products/primary-antibodies/phospho-acetyl-coa-carboxylase-ser79-antibody/3661),

anti-ACC antibody (CST, 3662S, https://www.cellsignal.com/products/primary-antibodies/acetyl-coa-carboxylase-antibody/3662) and
anti-GAPDH antibody (CST, 2118S, https://www.cellsignal.com/products/primary-antibodies/gapdh-14c10-rabbit-mab/2118) were used for human sample western. These applications were validated by their manufacturer.
anti-C4ORF48 antibody (Abcam, ab185315, https://www.abcam.com/c4orf48-antibody-ab185315-references.html#top-433) was used to detect a putative human secreted protein C4ORF48 in western. This antibody was not tested for this application. We detected a band at expected size in cell culture media which was abolished by protein secretion inhibitor BFA.
anti-HA antibody (BioLegend, 901502) was used to detect HA fusion proteins in western and immunostaining. These applications were validated by BioLegend (https://www.biolegend.com/en-us/products/purified-anti-ha-11-epitope-tag-antibody-11374).
anti-FLAG antibody (BioLegend, 637302) was used to detect FLAG fusion proteins in western. This application was validated by BioLegend (https://www.biolegend.com/en-us/products/purified-anti-dykddddk-tag-antibody-4905).
Anti-MTCYB antibody (Proteintech, 55090-1-AP) was applied for mouse samples in western. This application was validated by Proteintech (https://www.ptglab.com/products/CYTB-Antibody-55090-1-AP.htm).
anti-COX4 antibody (AbClonal, A10098, https://abclonal.com/catalog-antibodies/COXIVMonoclonalAntibody/A10098), anti-UQCRFS1 antibody (Abcam, Ab14746, https://www.abcam.com/uqcrfs1risp-antibody-5a5-ab14746.html), anti-UQCRH antibody (Abcam, ab134949, https://www.abcam.com/uqcrh-antibody-epr9039b-ab134949.html#top-345), anti-UQCRC2 antibody (Abclonal, A4181, https://abclonal.com/catalog-antibodies/UQCRC2PolyclonalAntibody/A4181) and
anti-UQCRB antibody (Sigma, HPA043060, https://www.sigmaaldrich.com/catalog/product/sigma/hpa043060?lang=en&region=SG) were used to detect proteins of interest in human, mouse, zebrafish samples in western. Their applications in western were validated for human and mouse by manufacturers. In zebrafish BN-PAGE western, these antibodies detected distinctive Complex IV (anti-COX4) or Complex III (anti-UQCRFA1, anti-UQCRH, anti-UQCRC2, anti-UQCRB) bands at expected sizes.
Anti-AMPK, 1:3000 (Bio-Rad, VMA00246) was applied to detect mouse AMPK in western. We detected a clear band at the expected size even though this antibody was not tested for mouse samples before. Anti-pAMPK, 1:3000 (CST, 2535) was applied to detect mouse AMPK in western, and this application was validated by CST (https://www.cellsignal.com/products/primary-antibodies/phospho-ampka-thr172-40h9-rabbit-mab/2535).
All the secondary antibodies were tested to have minimal cross reactivity to other species by their manufacturers. In immunofluorescence, the background of secondary antibody staining was tested.

Eukaryotic cell lines

Policy information about cell lines

Cell line source(s)	HEK293T, HeLa cells were obtained from ATCC. U87 MG cells were a gift of David Virshup's lab, Duke-NUS and were originated from ATCC HTB-14.
Authentication	Cell lines are show their typical morphology and doubling time. No additional authentication are performed.
Mycoplasma contamination	Cell lines are negative for mycoplasma as checked by MycoAlert kit, LONZA.
Commonly misidentified lines (See ICLAC register)	No commonly misidentified cell lines were used.

Animals and other organisms

Policy information about studies involving animals; ARRIVE guidelines recommended for reporting animal research

Laboratory animals	Zebrafish AB/Tuebingen was used in this study. Age of animal used was specified in figure legends. Mouse C57BL6/J line was used. 1-month-old female animals were used for AAV mediated BR expression .
Wild animals	This study does not involve wild animals.
Field-collected samples	Field-collected samples are not used in this study.
Ethics oversight	Zebrafish studies were performed under approved Institutional Animal Care and Use Committee (IACUC) protocols governed by A*STAR Singapore. Mouse studies were performed under protocol approved by Duke-NUS, SingHealth IACUC.

Note that full information on the approval of the study protocol must also be provided in the manuscript.
